# Association of ambient PM_2·5_ exposure with maternal bone strength in pregnant women from Mexico City: a longitudinal cohort study

**DOI:** 10.1016/S2542-5196(20)30220-5

**Published:** 2020-11

**Authors:** Haotian Wu, Marianthi-Anna Kioumourtzoglou, Allan C Just, Itai Kloog, Alison Sanders, Katherine Svensson, Nia McRae, Marcela Tamayo-Ortiz, Maritsa Solano-González, Robert O Wright, Martha M Téllez-Rojo, Andrea A Baccarelli

**Affiliations:** Department of Environmental Health Sciences, Mailman School of Public Health, Columbia University, New York, NY, USA (H Wu PhD, M-A Kioumourtzoglou PhD, Prof A A Baccarelli PhD); Department of Environmental Medicine and Public Health (A C Just PhD, A Sanders PhD, N McRae MPH, Prof R O Wright PhD) and Department of Pediatrics (A Sanders, Prof R O Wright), Icahn School of Medicine at Mount Sinai, New York, NY, USA; Department of Geography, Ben Gurion University of the Negev, Be’er Sheva, Israel (I Kloog PhD); Department of Health Sciences, Karlstad University, Karlstad, Sweden (K Svensson MS); Center for Nutrition and Health Research, National Institute of Public Health, Ministry of Health, Cuernavaca, Morelos, Mexico (M Tamayo-Ortiz ScD, M Solano-González MSc, Prof M M Téllez-Rojo PhD); and Mexican Council for Science and Technology, Mexico City, Mexico (M Tamayo-Ortiz)

## Abstract

**Background:**

Pregnancy is associated with deteriorations in maternal bone strength and heightened susceptibility to bone fractures. We aimed to investigate whether ambient particulate matter (PM)_2·5_ concentrations were associated with bone strength during pregnancy.

**Methods:**

In this longitudinal cohort study, we analysed longitudinal data from women participating in the Programming Research in Obesity, Growth, Environment and Social Stressors (PROGRESS) cohort in Mexico City, Mexico. Eligible women were aged 18 years or older, at less than 20 weeks’ gestation at the time of recruitment, planning to stay in Mexico City for the next 3 years, without heart or kidney disease, did not use steroids or anti-epileptic drugs, were not daily consumers of alcohol, and had access to a telephone. Daily ambient PM_2·5_ concentrations were estimated from a spatio-temporal model that was based on the individual’s address. Trabecular bone strength was measured using quantitative ultrasound from the radius of the middle finger and cortical bone strength from the proximal phalanx of the middle finger, during the second trimester, third trimester, and 1 and 6 months post partum. Bone strength T scores were modelled with PM_2·5_ concentrations using linear mixed models and distributed lag models.

**Findings:**

Adjusting for multiple exposure windows, each 10 ug/m³ increase in PM_2·5_ exposure concentrations in the first trimester was associated with a 0·18 SD decrease (95% CI −0·35 to −0·01; p=0·033) in ultrasound speed-of-sound (SOS) T score of trabecular bone strength from the second trimester until 6 months post partum. Similarly, each 10 μg/m³ increase in third trimester PM_2·5_ exposure was associated with a 0·18 SD decrease (−0·36 to −0·01; p=0·044) in the SOS T score of trabecular bone strength from the third trimester until 6 months post partum. PM_2·5_ exposure in the first month post partum was associated with a 0·20 SD decline (−0·39 to −0·01; p=0·043) in cortical bone strength until 6 months post partum.

**Interpretation:**

Ambient PM_2·5_ exposure during and after pregnancy was associated with diminished trabecular and cortical bone strength. Early pregnancy PM_2·5_ exposure was associated with a greater decline in bone strength later during pregnancy. Late pregnancy and early post-partum exposures adversely affected the post-partum bone strength recovery. Technological and policy solutions to reduce PM_2·5_ pollution could improve public health by reducing bone fracture risk.

## Introduction

Pregnancy is associated with changes in calcium homoeostasis that can lead to deteriorations in maternal bone density.^1^ Compared with preconception and early pregnancy periods, bone remodelling is more active during the second and third trimester when 2–3% of maternal bone calcium is transferred to the fetus for calcium-related growth and functioning,^2^ resulting in the loss of maternal bone mineral density (BMD).^2^ Although pregnancy-associated BMD loss physiologically recovers,^3^ the temporary decrease during pregnancy lowers bone strength and represents a period of heightened susceptibility to bone fractures and later life osteoporosis, particularly if co-exposures increase mineral loss or prevent full recovery. Thus, investigating modifiable environmental exposures that might exaggerate such susceptibility is of great public health interest.

Concentrations of fine particulate air pollution (particulate matter [PM]_2·5_) are rising globally, disproportionately affecting low-income and middle-income countries.^4^ PM_2·5_ exposure is a major global public health concern, associated with numerous adverse health outcomes.^5–10^ Evidence also suggests that PM_2·5_ might be associated with poor bone health. Studies of Norwegian men aged 75–76 years reported associations of ambient PM_2·5_ exposure with reduced total body BMD^11^ and increased risk of distal forearm fractures.^12^ A study of 10-year-old German children reported that PM_2·5_ was associated with serum osteocalcin and C-terminal telopeptide of type I collagen (CTX),^13^ two bone turnover markers that have been correlated with reduced bone density, increased risk of osteoporosis, and increased bone fracture risk.^14^ An analysis of US Medicare data showed an increased risk of hospital admissions related to bone fractures that were associated with ambient PM_2·5_ exposure, particularly within low-income communities.^15^ The same study analysed data from a cohort of men from the Greater Boston area, MA, USA, and reported associations of PM_2·5_ exposure with reduced serum parathyroid hormone, a key regulator of calcium metabolism, and that increased black carbon exposure was associated with decreased BMD at five anatomical sites.^15^

Pregnancy-associated changes in BMD and bone strength are expected, given the neonatal developmental needs. However, environmentally induced changes in bone strength might lead to an increased risk of fractures during pregnancy, particularly if these changes persist after the pregnancy period. To our knowledge, no study to date has examined the associations between ambient PM_2·5_ exposure and bone strength in pregnant women. To address this research gap, we aimed to investigate the association between maternal PM_2·5_ exposure and maternal bone strength using data from the Programming Research in Obesity, Growth, Environment and Social Stressors (PROGRESS) study based in Mexico City, Mexico.

## Methods

### Study design and participants

In this longitudinal cohort study, pregnant women from Mexico City, Mexico, receiving prenatal care from the Mexican Social Security Institute were recruited into PROGRESS. Eligible women were aged 18 years or older, at less than 20 weeks’ gestation at the time of recruitment, planning to stay in Mexico City for the next 3 years, without heart or kidney disease, did not use steroids or anti-epileptic drugs, were not daily consumers of alcohol, and had access to a telephone. Written informed consent was obtained from all participants. The study protocols were approved by institutional review boards at the Brigham and Women’s Hospital, Icahn School of Medicine at Mount Sinai, and the Mexican National Institute of Public Health.

### Procedures

#### PM_2·5_ exposure assessment

Daily ambient PM_2·5_ concentrations were estimated using a spatio-temporal model,^16^ starting 60 days before the estimated date of conception until 6 months post partum. In brief, daily, satellite-derived data on aerosol optical depth from the Moderate Resolution Imaging Spectro-radiometer at a 1×1 km spatial resolution were calibrated with local PM_2·5_ data from 12 monitoring stations, and integrated with land use variables and other spatial predictors (roadway density, temperature, relative humidity, planetary boundary layer, and daily precipitation) to predict daily PM_2·5_ concentrations. The prediction models were developed specifically for Mexico City, with a mean leave-one-out cross validation *r*^2^ of 0·72. Daily PM_2·5_ concentrations were averaged across five exposure periods, including preconception (defined as 60 days before conception), first trimester, second trimester, third trimester, and 1 month post partum.

#### Bone strength assessment

Quantitative ultrasound, a non-invasive method of estimating bone strength in the peripheral skeleton,^17^ was administered by a trained nurse during four visits (at the second trimester [16–22 weeks’ gestation], third trimester [27–36 weeks’ gestation], 1 month post partum, and 6 months post partum) using a Sunlight Omnisense 7000 bone sonometer (BeamMed, Plantation, FL, USA). The velocity of the ultrasound transmission reflects a combination of bone density, architecture, and elasticity and is an important predictor of fracture risk.^18^ For all visits, the ultrasound scans were taken for the radius and the proximal phalanx of the middle finger, which reflect trabecular and cortical bones, respectively. The speed-of-sound (SOS) measurements were converted to T scores using a machine-provided standard population that was age specific and sex specific.

### Statistical analysis

To estimate the relationship between PM_2·5_ concentrations and maternal bone strength, linear mixed models including random intercepts per individual were fitted to account for the repeated measurement of bone SOS T scores at four stages (second trimester, third trimester, 1 month post partum, and 6 months post partum). Given that BMD is known to decline in pregnancy and recover during the post-partum period, time interval variables between visits (eg, second to third trimester) and the estimated gestation day at the study visit were included in the model as covariates to estimate and control for the natural changes in bone strength between visits. Interaction terms between PM_2·5_ concentrations and time intervals (which represent the changes in bone strength) were used to estimate the influence of PM_2·5_ on the natural trajectory of bone strength fluctuations over the visit intervals. We imposed temporality requirements for each model, ensuring that no model included exposures that occurred after the date of clinic visit and bone ultrasound. For example, models that included second trimester bone ultrasound T scores also included PM_2·5_ exposures up to, but not past, the day of second trimester visit.

Distributed lag models^19^ (DLMs) of daily PM_2·5_ exposures were used to assess temporal trends in the observed associations, identify potential important windows of susceptibility, and to examine the consistency of associations at each outcome assessment. DLMs were modelled using the dlm^20^ package in R (version 1.1.5).

We considered maternal age, body-mass index (BMI), gestation day of visit, education (less than high school, high school, further than high school), socioeconomic status (SES; calculated using the Mexican Association of Research and Public Opinion Agencies [AMAI] guidelines,^21^ collapsed into three categories for modelling), parity (0, 1, 2, >2), alcohol use (binary), smoking (binary), environmental smoke exposure at home (binary), calcium intake (continuous), vitamin D intake (continuous), seasonality of each visit (November–February, March–April, May–October), and date of enrolment as potential covariates. Selection for multivariable models was based on biological plausibility and statistical significance in the bivariate models (appendix p 4). Maternal age, BMI, gestation day at time of the visit, SES, education, and parity were included in all multivariable models. All other variables were not included in the multivariable model, but their influence on the PM_2·5_ and maternal bone strength relation were assessed in sensitivity analyses. We also examined potential effect modification by breastfeeding because breastfeeding is known to increase bone resorption. Additional methodological details can be found in the appendix (pp 5, 6).

### Role of the funding source

The funder of the study had no role in study design, data collection, data analysis, data interpretation, or writing of the report. HW and AAB had full access to all the data in the study and had final responsibility for the decision to submit for publication.

## Results

Between July 3, 2007, and Feb 21, 2011, we recruited 948 PROGRESS participants who had livebirths. Of these, 941 (99%) had PM_2·5_ exposure data, and 930 (98%) had bone ultrasound data from at least one visit, and one woman who gave birth before 28 weeks was excluded. A summary of demographic and lifestyle factor data is presented in the appendix (p 2). At the time of recruitment, the mean age of participants was 27·3 years (SD 5·5) and mean BMI was 26·9 kg/m^2^ (4·2). Most participants had not studied beyond high school, were not exposed to environmental smoke exposure at home, and did not consume alcohol during pregnancy (appendix p 2). The study population was generally of low SES, with 698 (74%) individuals in the bottom three categories of the AMAI index. PM_2·5_ concentrations and T scores of bone strength ultrasounds are presented in table 1 and the appendix (p 3). PM_2·5_ exposure was consistent with previous estimates of PM_2·5_ levels in Mexico City^16^ and higher when compared with the estimated Mexican national average (estimated 13·04 μg/m³ in 2010).^22^

Table 2 presents the overall associations of PM_2·5_ exposure concentrations with SOS T scores of maternal bone strength. Adjusting for maternal age, BMI, SES, education, parity, gestation time, and PM_2·5_ concentrations during the 60 days before conception and during the second trimester, each 10 μg/m³ increase in first trimester PM_2·5_ exposure was associated with a 0·18 decrease (95% CI −0·35 to −0·01; p=0·033) in SOS T score of trabecular bone from the second trimester until 6 months post partum. Similarly, each 10 μg/m³ increase in third trimester PM_2·5_ exposure was associated with a 0·18 decrease (−0·36 to −0·01; p=0·044) in the SOS T score of trabecular bones from the third trimester until 6 months post partum (table 2). PM_2·5_ exposure in the first month post partum was associated with a decreased SOS T score of cortical bone (β=−0·20 [95% CI −0·39 to −0·01]; p=0·043) at 1 and 6 months post partum (table 2).

**Figure 1:**
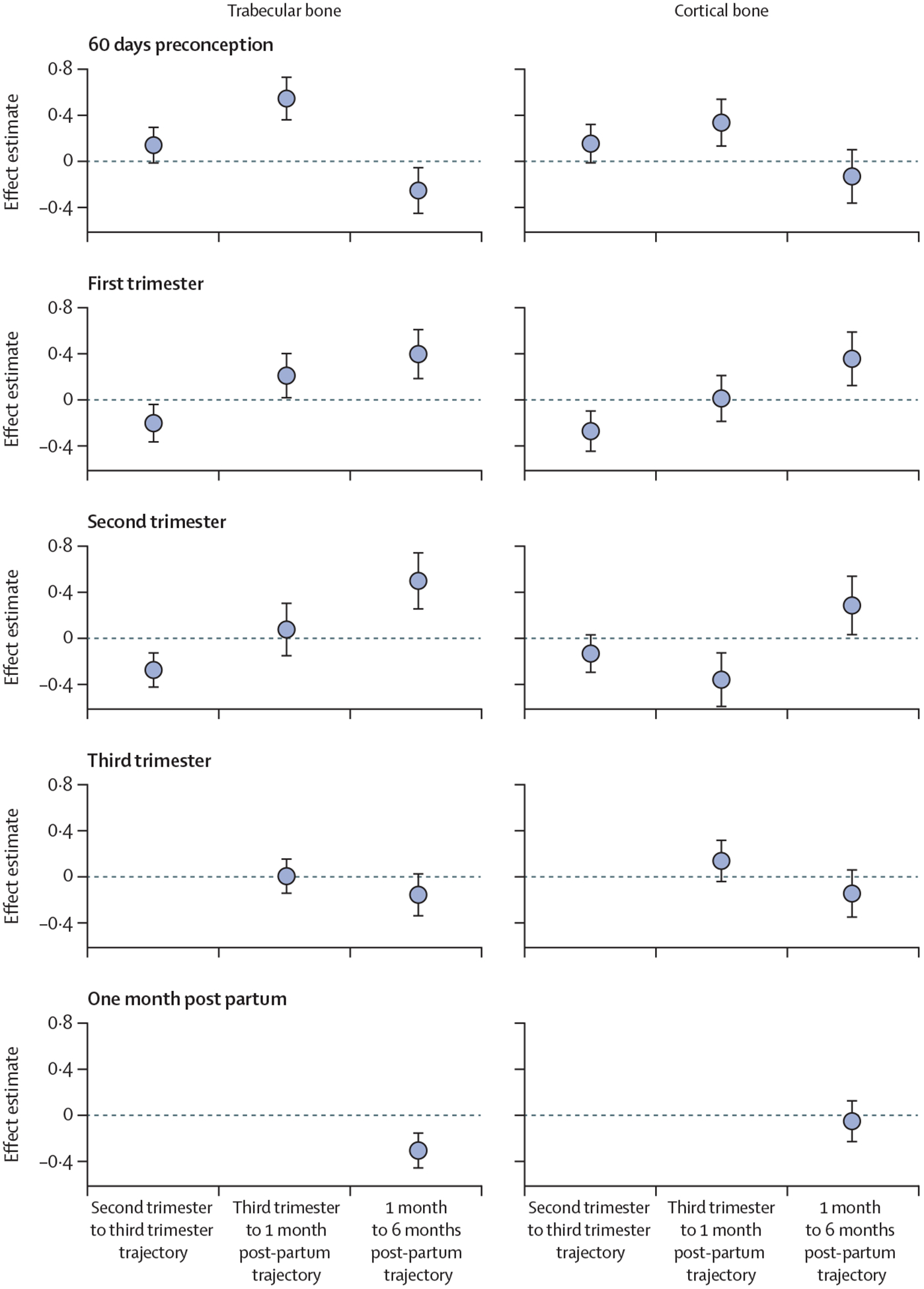
The influence of stage-specific PM_2·5_ exposure on the natural trajectory of bone strength changes during and after pregnancy The effect estimate (regression β) and corresponding 95% CIs are from interaction terms between PM_2·5_ concentrations and the estimated change in speed-of-sound T score, expressed as additional T score change per 10 μg/m³ increase in PM_2·5_ exposure. Positive associations represent higher than expected trajectory of bone strength (ie, decreased negative trajectory during pregnancy or increased positive trajectory during the post-partum period) related to higher PM_2·5_ concentrations. Conversely, inverse associations represent lower than expected bone strength, and can be interpreted as increased negative trajectory during pregnancy or decreased positive trajectory during the post-partum period. All models were adjusted for maternal age, body-mass index, socioeconomic status, education, parity, and PM_2·5_ concentrations at other time periods. PM=particulate matter.

We used interaction terms between PM_2·5_ exposure concentrations and each time interval between visits to assess temporal trends in the associations between PM_2·5_ exposure and maternal bone strength. Figure 1 and the appendix (pp 5, 6) show generally consistent temporal trends in the associations between PM_2·5_ exposure and bone strength for trabecular and cortical bones. Mean preconception PM_2·5_ exposure was positively associated with the SOS T score trajectories of trabecular and cortical bones between the second and third trimesters as well as between the third trimester and 1 month post partum, indicating that higher preconception PM_2·5_ exposure was associated with less maternal bone strength decline during mid-to-late gestation. First and second trimester PM_2·5_ exposures were both inversely associated with SOS T score trajectories of trabecular and cortical bones between the second and third trimesters, but association reversed over time, as indicated by the positive associations between first and second trimester PM_2·5_ concentrations and the bone SOS T score trajectory between 1 and 6 months post partum. These time-specific findings indicate that first and early second trimester PM_2·5_ exposure was associated with greater maternal bone strength decline during pregnancy as well as increased post-partum bone strength recovery. PM_2·5_ exposure concentrations for the third trimester and the first month post partum were both inversely associated with the SOS T score trajectories of trabecular and cortical bones between 1 and 6 months post partum, which suggests that for women with high third trimester and first month post-partum PM_2·5_ exposures, the expected rate of maternal post-partum bone strength recovery is diminished (figure 1; appendix pp 5, 6).

**Figure 2:**
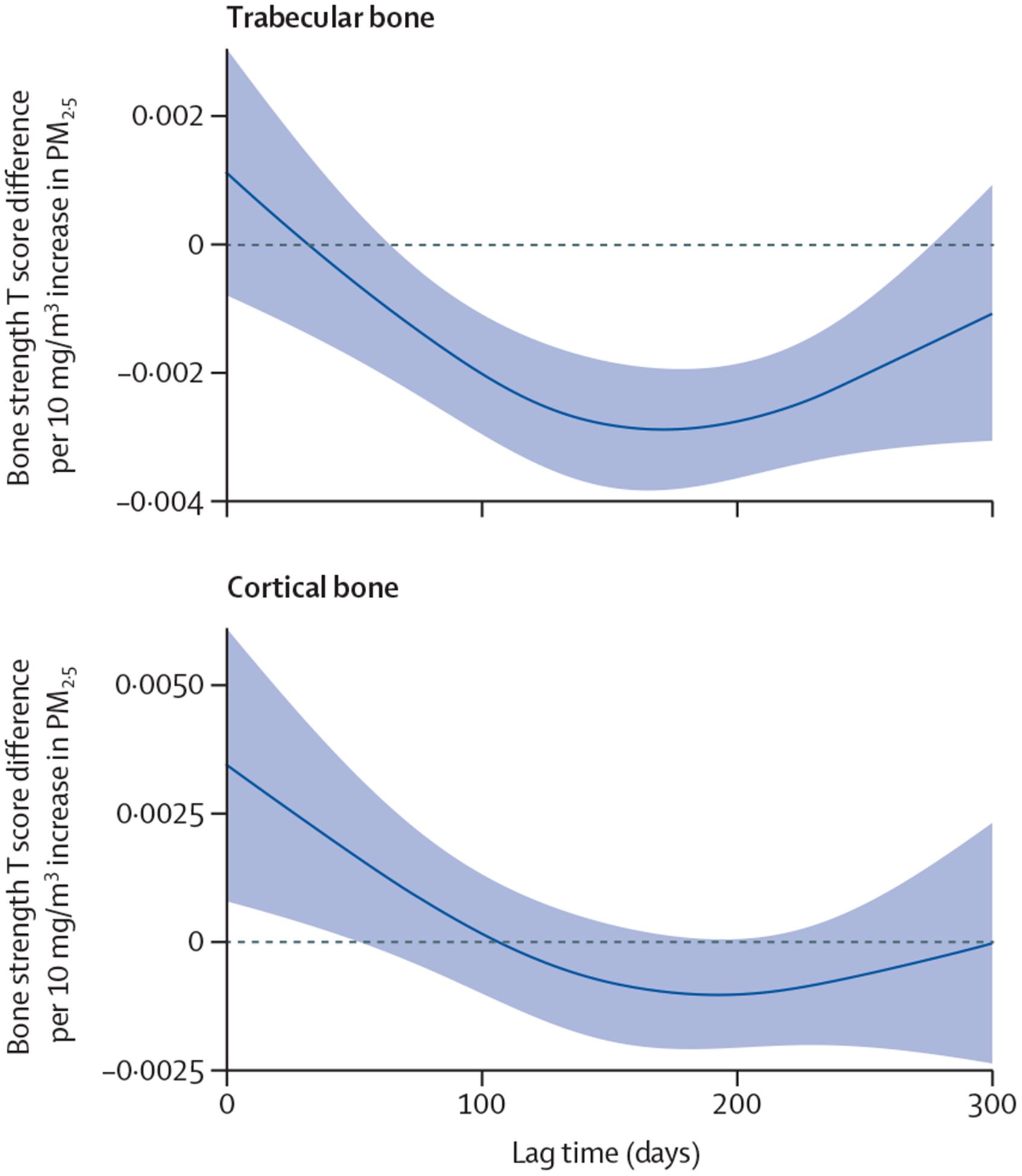
Distributed lag model of ultrasound speed-of-sound T score differences per unit increase in daily PM_2·5_ exposure (μg/m³) Lag day 0 represents the day of bone ultrasound and lag day 300 represents the 300th day before the day of bone ultrasound. PM=particulate matter.

One major observation from the trajectory interaction models shown in figure 1 is the consistent time-varying association between PM_2·5_ concentration and bone strength. In models that controlled for exposure at other periods, PM_2·5_ concentrations were inversely associated with bone strength trajectories in the time periods immediately following exposure, but a positive association, indicating recovery, was observed after (figure 1). Given this observation, DLMs using daily PM_2·5_ data were used to examine the relationship between daily PM_2·5_ con centrations and maternal bone SOS T scores up to 300 days before measurement. Figure 2 shows DLMs considering all bone strength measurements and adjusting for the natural trajectory of bone strength throughout the follow-up period. For cortical bones, we found a downward trajectory, reaching the nadir at 190 days, but no statistically significant inverse association was observed at any point (figure 2). For trabecular bones, we found a statistically significant inverse association between PM_2·5_ concentrations and bone strength from days 65 to 275 (figure 2). The timing and shape of the trajectory were reasonably similar when each stage was considered individually (appendix pp 7–10), with some minor differences for second trimester outcomes.

The addition of calcium intake, vitamin D intake, alcohol, environmental smoke exposure at home, date of enrolment, and seasonality variables did not meaningfully change the effect estimates and observed trends (data not shown). Additionally, we did not observe any notable differences in effect estimates when stratified by breastfeeding status or when those who reported smoking during pregnancy (n=6) were excluded.

## Discussion

We found evidence that ambient PM_2·5_ exposures were associated with changes in maternal bone strength. We found a stronger association for trabecular bone compared with cortical bone, but the directions of association and temporal patterns were similar. In general, high ambient PM_2·5_ exposure was associated with deteriorations in bone strength that were observable after 2–3 months’ lag time, although this association did not persist over long lag periods. Although bone density and bone strength loss are expected during pregnancy, particularly during the second and third trimester when the mother supports a growing fetal skeleton, our results suggest that long-term ambient PM_2·5_ exposure exacerbates the loss of bone strength during pregnancy. Accordingly, although patterns of association were similar during pregnancy and post partum, ambient PM_2·5_ pollution transiently exaggerates bone strength loss during pregnancy and might lead to increased risk for osteoporosis and bone fractures during pregnancy and post partum.

The inverse associations observed between ambient PM_2·5_ exposure and bone strength during pregnancy and post partum are generally consistent with results from previous studies of fine particulate air pollution and BMD. Two Norwegian studies reported that higher long-term PM_2·5_ exposure was associated with lower total body BMD, particularly among former and current smokers,^11^ and higher risk of forearm fractures in older men.^12^ In the USA, higher ambient PM_2·5_ exposure was associated with increased risk of bone fracture hospital admissions.^15^ Black carbon, a major component of PM_2·5_ associated with traffic emissions, was specifically found to be associated with BMD loss at five different anatomical sites.^15^

A unique feature of our study is the use of repeated bone ultrasounds to measure bone strength instead of dual-energy x-ray absorptiometry (DEXA) used in previous studies. DEXA assesses only the mineral components and strictly reflects BMD. By contrast, bone ultrasound is influenced by the mineral and organic components and measures bone strength as a combination of BMD, bone microarchitecture, and elasticity.^17^ Given that previous studies have associated ambient PM_2·5_ exposure with reduced BMD, as measured by DEXA, the decrease in bone ultrasound SOS T scores observed in our study is probably, at least partially, due to decreased BMD. Deteriorations in the mineral component of the bone would be consistent with our observation that higher PM_2·5_ concentrations are associated with increased pregnancy-related bone strength loss, which is typically from the mineral component.^2^ However, the associations between bone ultrasound SOS and PM_2·5_ concentrations could be reflective of changes in the organic matrix of the bone or other bone characteristics such as microarchitecture and elasticity.

Although which tissue types and bone properties are affected by ambient PM_2·5_ exposure is unclear, any changes measured by bone ultrasound SOS probably also imply changes in bone remodelling and turnover. This conclusion is strengthened by the observation that our results suggest that PM_2·5_ concentrations have a stronger influence on trabecular bone than cortical bone, which might be due to trabecular bone being more metabolically active than cortical bone. The non-monotonic pattern of associations across time observed in our study need to be replicated by independent studies, but could indicate increased bone turnover. Typically, bone remodelling undergoes bone resorption followed by bone formation, which under optimal conditions takes approximately 10 days and 3 months, respectively.^23^ This bone remodelling sequence and relative timing is consistent with our models, in which deteriorations of bone strength were associated with exposure to PM_2·5_ concentrations 2–3 months earlier, while gains in bone strength were associated with exposure to PM_2·5_ concentrations greater than 6 months earlier. This non-monotonicity indicates that ambient PM_2·5_ exposure could be associated with both bone resorption and formation. Previous studies have reported positive associations of PM_2·5_ exposure with serum parathyroid hormone^15^ and osteocalcin,^13^ two biomarkers of bone formation, as well as CTX,^13^ a biomarker of bone resorption. Whether transiently increased bone turnover and remodelling are associated with long-term bone health beyond potentially increased risk of bone fractures is unclear.

The exact biological mechanisms by which ambient PM_2·5_ exposure affects bone remodelling factors are unknown, but our results suggest that the mechanism might not be exclusive to pregnancy. All models were mutually adjusted for PM_2·5_ exposures at other stages, reducing the likelihood of confounding from correlated exposures. We found that PM_2·5_ exposures during the third trimester and first month post partum are inversely associated with post-partum bone strength recovery, which is consistent with the inverse-then-positive pattern of maternal bone strength as observed for first and second trimester PM_2·5_ exposures. If the observed relationships were exclusive to pregnancy, one might expect either no association between PM_2·5_ concentrations in the first month post partum and post-partum bone strength recovery or a positive association, similar to that for first and second trimester PM_2·5_ exposures. Thus, our results, combined with those of previous studies of ambient PM_2·5_ exposure and BMD in non-pregnant populations, suggest some of the underlying biological mechanisms of the relationship between ambient PM_2·5_ exposure and maternal bone strength are not pregnancy specific. Accumulating evidence suggests that oxidative stress and inflammation might play a role and that PM pollution causes oxidative damage^24^ and systemic inflammation,^25^ both of which promote bone resorption and inhibit bone growth.^26^ Ambient PM_2·5_ exposure could disrupt hormonal regulators of bone remodelling because PM pollution has been linked to the disruption of sex hormones^27^ and several hormones are systemic regulators of bone remodelling.^28^

All examined periods of PM_2·5_ exposures exhibited similar time-varying patterns in their associations with bone strength. However, time of exposure is associated with different health consequences. Accounting for lag time and the natural bone strength decline during the second and third trimesters, increased ambient PM_2·5_ exposure in the first or early second trimester is expected to result in increased bone strength loss later in pregnancy, exacerbating this period of susceptibility to bone fractures. By contrast, exposure during the third trimester or shortly following birth will lead to hindrance of the normal post-partum bone recovery, potentially extending the window of susceptibility to bone fractures longer into post partum.

Our study has limitations. First, non-differential exposure-measurement error probably occurred because individual PM_2·5_ concentrations were estimated on the basis of home addresses and spatio-temporal prediction models might not perfectly capture the individual’s exposure for any given day. However, proxy exposures are less susceptible compared with personal exposures to reverse causation and biases from confounding by hard-to-control personal factors such as behaviour.^29^ Furthermore, mean PM_2·5_ concentrations across five periods over 1 year are probably able to capture long-term exposure patterns. Non-differential exposure-measurement error usually biases the effect estimates toward the null because no reason exists to believe that any error in PM_2·5_ predictions is related to bone strength.^30^ Despite the exposure measurement error, we were able to identify statistically significant associations of PM_2·5_ exposure with bone strength. Another limitation is the absence of data on physical activity, which might be correlated with PM_2·5_ concentrations and bone strength. Although we cannot exclude the possibility of residual confounding from exercise or other factors, we do not expect our results to be fully attributed to residual confounding. Lastly, our sample of 930 pregnant women from Mexico City lacks generalisability to other populations. In addition to expected differences in sociodemographic factors between our study population and others, the compositions of ambient PM_2·5_ in different communities vary on the basis of the sources of pollution.

To our knowledge, this is the first study to examine the associations of ambient PM_2·5_ pollution with bone strength in pregnant women. The prospective design and repeated measurements of PM_2·5_ exposure and bone strength are unique to the PROGRESS cohort and allowed us to capture the influence of PM_2·5_ exposure on the trajectory of bone strength changes during pregnancy and 6 months post partum and also to assess the timing of this relationship.

The specific temporal patterns suggest that recovery might occur if later exposures are mitigated or minimised. In the context of pregnancies, high ambient PM_2·5_ exposure early in pregnancy might lead to higher risk of bone fractures during mid-to-late pregnancy. The hindrance of the normal post-partum bone strength recovery period might be due to effect modification with pregnancy-related changes in bone strength, but could be an independent effect of PM_2·5_ as well, unrelated to pregnancy. Given the high ambient PM_2·5_ found in many areas globally, particularly in low-income communities, innovation in technology and policy that seeks to reduce pollutant levels could improve public health by reducing bone fracture risk.

## Supplementary Material

1

## Figures and Tables

**Table 1:** Bone strength ultrasound T scores of pregnant women enrolled in the PROGRESS cohort

	Cortical bone (from the radius; n=941)	Trabecular bone (from proximal phalanx on the middle finger; n=941)
Second trimester	893 (95%); −1·5 (1·2)	814 (87%); −0·5 (1·1)
Third trimester	720 (77%); −1·6 (1·1)	643 (68%); −0·6 (1·2)
1 month post partum	603 (64%); −1·5 (1·1)	496 (53%); −0·5 (1·1)
6 months post partum	480 (51%); −1·5 (1·1)	410 (44%); −0·6 (1·2)

Data are n (%) or mean (SD). T scores were based on an internal reference population of non-pregnant, white, North American adults, stratified by sex and age. Progress=Programming Research in Obesity, Growth, Environment and Social Stressors.

**Table 2:** Associations of bone ultrasound speed-of-sound T scores with PM_2·5_ exposures before, during, and after pregnancy

	Trabecular bone strength T score		Cortical bone strength T score	
	β (95% CI)*	p value	β (95% CI)*	p value
60 days preconceptionf	−0·05 (−0·20 to 0·11)	0·56	0·04 (−0·13 to 0·22)	0·65
First trimester†	−0·18 (−0·35 to −0·01)	0·033	−0·06 (−0·26 to 0·13)	0·53
Second trimester‡	0·09 (−0·08 to 0·26)	0·32	0·06 (−0.11 to 0.23)	0·48
Third trimester§	−0·18 (−0·36 to −0·01)	0·044	−0·08 (−0·31 to 0·15)	0·48
First month post partum§	0·03 (−0·11 to 0·17)	0·67	−0·20 (−0·39 to −0·01)	0·043

PM=particulate matter.

* Expressed as SD change per 10 ug/m^3^ increase in PM_2·5_. All models were adjusted for maternal age, body-mass index, socioeconomic status, education, parity, time since conception, natural trajectory of bone strength changes over time, and PM_2·5_ concentrations at other stages.

† Model included outcomes at second trimester, third trimester, 1 month post partum, and 6 months post partum.

‡ Model included outcomes at third trimester, 1 month post partum, and 6 months post partum.

§ Model included outcomes at 1 and 6 months post partum

## Research in context

### Evidence before this study

We searched PubMed and Web of Science for studies related to particulate matter (PM) air pollution and bone density, published in English from database inception until June 30, 2019. We used a combination of search terms, including “PM2·5”, “fine particulate matter”, “bone strength”, “DEXA”, “bone density”, and other related terms. Pregnancy is associated with transient deteriorations in maternal bone strength and susceptibility to bone fractures. PM_2·5_ has been associated with bone mineral density, but to our knowledge, whether ambient PM_2·5_ concentrations are associated with bone strength during this life stage has not been addressed.

### Added value of this study

Ambient PM_2·5_ exposure during and after pregnancy was associated with diminished trabecular and cortical bone strength, with slightly stronger effects observed for trabecular bones than for cortical bones. Specifically, high ambient PM_2·5_ exposure is associated with deteriorations in bone strength observable after 2–3 months’ lag time, although this association is attenuated over time. This time-course indicates that recovery might occur if later exposures are mitigated or minimised. High ambient PM_2·5_ exposure early in pregnancy was associated with exaggerated pregnancy-related bone strength loss, which is likely to lead to an increased risk of bone fractures during mid-to-late pregnancy. Exposures in late pregnancy or early post partum were associated with hindrance of the normal post-partum bone strength recovery period, which might extend the window of susceptibility for bone fractures. To our knowledge, this is the first study to examine the associations of ambient PM_2·5_ pollution with bone strength in pregnant women and to examine the temporal trends of the associations.

### Implications of all the available evidence

Our study of ambient PM_2·5_ pollution and bone strength in pregnant Mexican women, combined with evidence from previous studies of ambient PM_2·5_ pollution and bone mineral density in other populations, suggests that ambient PM_2·5_ pollution is associated with changes in bone remodelling and turnover. Although we found no indications of a specific period of susceptibility, the temporal patterns of the relationship observed in our study suggest that the bone strength loss might not be permanent if PM_2·5_ exposure is mitigated or minimised. Technology and policies that seek to minimise PM_2·5_ pollutant concentrations could mitigate the public health effects by reducing bone fracture risk.
