# Supplementary material for: Association of ambient PM2·5 exposure with maternal bone strength in pregnant women from Mexico City: a longitudinal cohort study
Source: Lancet Planet Health. Author manuscript; Available in PMC 2020 Nov 13. (PMC7664993; doi:10.1016/S2542-5196(20)30220-5)
Supplement: 1 [file NIHMS1644204-supplement-1.pdf]

THE LANCET
Planetary Health

Supplementary appendix

This appendix formed part of the original submission and has been peer reviewed. We post it as supplied by the authors.

Supplement to: Wu H, Kioumourtzoglou M-A, Just AC, et al. Association of ambient $PM_{2·5}$ exposure with maternal bone strength in pregnant women from Mexico City: a longitudinal cohort study. *Lancet Planet Health* 2020; **4:** e530–37.

Supplemental Information

Table of Contents

Page 2. Demographics

Supplemental Table 1. Demographics of the PROGRESS cohort.

Page 3. Summary Exposure Data

Supplemental Table 2. Demographics of the PROGRESS cohort.

Page 4. Additional Information on Covariate Selection

Supplemental Table 3. Bivariate Models of Potential Covariates with Exposure and Outcomes

Pages 5-6. Additional Information and Data on Piecewise Trajectory Mixed Models

Supplemental Table 4. Interaction estimates between trabecular bone ultrasound speed of sound t-scores with $PM_{2.5}$ exposures.

Supplemental Table 5. Interaction estimates between cortical bone ultrasound speed of sound t-scores with $PM_{2.5}$ exposures.

Pages 7-10. Additional Information and Data on Distributed Lag Models

Supplemental Figure 1. Distributed lag model of trabecular bone ultrasound speed of sound t-score differences per unit increase in daily PM2.5 exposure (µg/m^3).

Supplemental Figure 2. Distributed lag model of cortical bone ultrasound speed of sound t-score changes per unit increase in daily PM2.5 exposure (µg/m^3).

Demographics of the PROGRESS cohort

Age, years	27·3 (5·5)
BMI, kg/m²	
Second trimester	941 (100%); 26·9 (4·2)
Third trimester	790 (84%); 29·4 (4·1)
1 month post partum	687 (73%); 27·1 (4·0)
6 months post partum	630 (67%); 26·9 (4·6)
Socioeconomic status index*	
1 (lowest)	84 (9%)
2	398 (42%)
3	216 (23%)
4	137 (15%)
5	88 (9%)
6 (highest)	18 (2%)
Education	
<High school	383 (41%)
High school	331 (35%)
>High school	227 (24%)
Parity	
0	425 (45%)
1	379 (40%)
2	121 (13%)
≥3	16 (2%)
Exposure to environmental tobacco smoke at home	
No	639 (68%)
Yes	295 (32%)
Current alcohol consumption at second trimester	
No	766 (97%)
Yes	25 (3%)
Breastfeeding at 1 month post partum	
Never	51 (7%)
Attempted but did not sustain	47 (6%)
Non-exclusive	468 (61%)
Exclusive breastfeeding	202 (26%)

Data are n (%) or mean (SD). BMI=body-mass index. *Calculated using the characteristics of the household according to using the Mexican Association of Research and Public Opinion Agencies criteria and collapsed into three categories for modelling.

Exposure Summary Data

**Supplemental Table 2. Summary of $PM_{2.5}$ and Bone Strength Ultrasound T-scores Data of Pregnant Women Enrolled in the PROGRESS Cohort**				
		N	Mean	SD
$PM_{2.5}$ Exposure (µg/cm^3)	60 Days Preconception	941	23·5	4·6
	First Trimester	941	22·7	4·3
	Second Trimester	941	22·3	4·2
	Third Trimester	941	23·1	5·1
	1 Month Postpartum	941	23·5	5·8
	6 Months Postpartum	941	22·7	3·6

Additional Information on Covariate Selection

Bivariate relations of $PM_{2.5}$ and maternal bone strength with potential covariates were assessed using Pearson correlation tests, t-tests, and one-way ANOVA tests. Results are presented in Appendix A (Supplemental Table 1). Results from Spearman rank correlations, Wilcoxon rank sum tests, and Kruskal-Wallis tests were similar.

Supplemental Table 3. Bivariate Correlations Between Potential Covariates with PM2.5 and Bone Strength

	Pregnancy Average PM2.5		Bone Strength (2T)			
			Radial		Cortical	
	Correlation Coefficient	p-value	Correlation Coefficient	p-value	Correlation Coefficient	p-value
**Maternal Age (Year)**	0.03	0.33	0.24	0.00	-0.07	0.05
**BMI (kg/m2)**	0.03	0.40	-0.05	0.11	-0.12	0.00
**Gestation (day at visit)**	-0.42	0.00	0.05	0.11	-0.02	0.61
Calcium Intake (mg/day)*	0.02	0.67	-0.06	0.25	0.01	0.84
Vitamin D Intake (IU/day)*	-0.06	0.41	0.04	0.57	-0.02	0.84
**Socioeconomic Status**	Mean	p-value	Mean	p-value	Mean	p-value
**Low**	22.46	0.02	-1.49	0.35	-0.44	0.57
**Medium**	22.96		-1.52		-0.50	
**High**	22.77		-1.32		-0.57	
**Education**						
**<High School**	22.37	0.01	-1.40	0.07	-0.53	0.51
**High School**	22.97		-1.60		-0.43	
**>High School**	22.79		-1.43		-0.44	
**Parity (previous)**						
**0**	22.76	0.55	-1.65	0.00	-0.47	0.62
**1**	22.57		-1.36		-0.45	
**>1**	22.74		-1.28		-0.57	
Season						
Cold-dry (Nov-Feb)	22.12	<0.001	-1.42	0.16	-0.50	0.88
Warm-dry (Mar-Apr)	22.19		-1.63		-0.45	
Rainy (May-Oct)	23.23		-1.46		-0.46	
Environmental Tobacco at Home						
No	22.60	0.23	-1.45	0.25	-0.50	0.53
Yes	22.82		-1.54		-0.45	
Alcohol Use						
No	22.60	0.00	-1.48	0.90	-0.47	0.43
Yes	23.41		-1.46		-0.56	

*Both measures were taken at 3T. Results shown are for 3T bone strength.
Bolded variables were included in the models presented in the main manuscript

Variables not included in the models were assessed in sensitivity analyses. Ultimately, adjustment for these additional factors did not appreciably change the model estimates.

Additional Information and Data on Piecewise Trajectory Mixed Models

To estimate the relations between $PM_{2.5}$ levels and maternal bone strength, linear mixed models specifying random intercepts and fixed effects were fitted.

- The exposures were average $PM_{2.5}$ exposure at the specified periods – preconception (60 days before estimated day of conception), first trimester, second trimester, third trimester, and one month postpartum.
- The outcomes are bone SOS t-scores at four timepoints – second trimester, third trimester, 1 month postpartum, and 6 months postpartum).
- Because BMD is known to decline in pregnancy and recover postpartum, piecewise growth curve models with time variables (visits) were fitted to estimate and adjust for the trajectory between each ultrasound measurement (second to third trimester, third trimester to 1 month postpartum, and 1 to 6 months postpartum).

To maintain temporality, three separate models were built (all models were adjusted for the selected covariates):

- Model 1 –
  - $PM_{2.5}$ exposure from preconception, first trimester, and second trimester.
    - Second trimester exposure includes only the time until day of visit, not the entire trimester
  - Bone SOS t-scores from all four visits as the outcome.
  - This model was used to obtain the effect estimates for preconception and first trimester
- Model 2 –
  - $PM_{2.5}$ exposure from preconception, first trimester, second trimester, and third trimester.
    - Third trimester exposure includes only the time until day of visit, not the entire trimester
  - Bone SOS t-scores from three visits (T3, 1 month postpartum, and 6 months postpartum).
  - This model was used to obtain the effect estimates for second trimester
- Model 3 –
  - $PM_{2.5}$ exposure from preconception to 1 month postpartum
    - Postpartum exposure includes only the time until day of visit, not the entire trimester
  - Bone SOS t-scores from two visits (1 and 6 months postpartum).
  - This model was used to obtain the effect estimates for third trimester and first month postpartum

To assess the impact of $PM_{2.5}$ levels on the trajectory of bone strength changes, we added interaction terms between $PM_{2.5}$ levels and time variables. The time variables in our model represent the trajectory of change over time, including the loss of bone strength during pregnancy and the slow recovery postpartum.

In Supplemental Tables 2 and 3, we show model estimates corresponding to Figure 1 in the main manuscript. A positive interaction estimate represents that those who are exposed to higher ambient $PM_{2.5}$ levels have a higher than expected change during the indicated phase. Vice-versa for negative interaction estimates. In other words:

- During pregnancy – a positive interaction estimate represents less bone strength loss (associated with higher ambient $PM_{2.5}$ exposure)
- Postpartum – a positive interaction estimate represents greater bone strength gain/recovery (associated with higher ambient $PM_{2.5}$ exposure)

**Supplemental Table 4. Interaction estimates between trabecular bone ultrasound speed of sound t-scores with $PM_{2.5}$ exposures.**									
	**INTERACTION TERMS* (PM2.5 and Time Variable)**								
	Between Second and Third Trimester			Between third trimester and 1 month postpartum			Between 1 and 6 months postpartum		
Exposure Time	Estimate[1]	95% CI[1]	p-value	Estimate[1]	95% CI	p-value	Estimate[1]	95% CI[1]	p-value
60 Days Preconception[2]	0·14	-0·03, 0·30	0·097	0·55	0·36, 0·74	<0·001	-0·26	-0·47, -0·05	0·014
First Trimester[2]	-0·20	-0·37, -0·04	0·017	0·21	0·01, 0·40	0·041	0·39	0·17, 0·61	<0·001
Second Trimester[3]	-0·28	-0·44, -0·13	<0·001	0·07	-0·16, 0·31	0·551	0·50	0·24, 0·75	<0·001
Third Trimester[4]	NA			<0·001	-0·15, 0·16	0·997	-0·16	-0·35, 0·03	0·093
First Month Postpartum[4]	NA			NA			-0·29	-0·45, -0·14	<0·001

*Time indicator variable is used in this piecewise trajectory model to estimate the rate of change in bone strength (SOS t-score).
1Expressed as per 10 ug/m3 increase in $PM_{2.5.}$ All models adjusted for maternal age, BMI, SES, education, parity, time since conception, natural trajectory of bone strength changes over time, and exposure at other time periods.
2 Model includes outcomes at second trimester, third trimester, 1 month postpartum, and 6 months postpartum.
3 Model includes outcomes at third trimester, 1 month postpartum, and 6 months postpartum.
4 Model includes outcomes at 1 and 6 months postpartum.

**Supplemental Table 5. Interaction estimates between cortical bone ultrasound speed of sound t-scores with PM2.5 exposures.**									
	**INTERACTION TERMS* (PM2.5 and Time Variable)**								
	Between Second and Third Trimester			Between third trimester and 1 month postpartum			Between 1 and 6 months postpartum		
Exposure Time	Estimate[1]	95% CI[1]	p-value	Estimate[1]	95% CI[1]	p-value	Estimate[1]	95% CI[1]	p-value
60 Days Preconception[2]	0·15	-0·02, 0·32	0·085	0·33	0·12, 0·54	0·002	-0·13	-0·37, 0·11	0·278
First Trimester[2]	-0·27	-0·45, -0·09	0·003	0·01	-0·20, 0·21	0·930	0·35	0·11, 0·59	0·004
Second Trimester[3]	-0·13	-0·30, 0·04	0·125	-0·36	-0·60, -0·12	0·003	0·28	0·02, 0·54	0·032
Third Trimester[4]	NA			0·14	-0·01, 0·03	0·150	-0·15	-0·36, 0·07	0·174
First Month Postpartum[4]	NA			NA			-0·06	-0·24, 0·13	0·550

*Time indicator variable is used in this piecewise trajectory model to estimate the rate of change in bone strength (SOS t-score).
1 Expressed as per 10 ug/m^3 increase in $PM_{2.5.}$ All models adjusted for maternal age, BMI, SES, education, parity, time since conception, natural trajectory of bone strength changes over time, and exposure at other time periods.
2 Model includes outcomes at second trimester, third trimester, 1 month postpartum, and 6 months postpartum.
3 Model includes outcomes at third trimester, 1 month postpartum, and 6 months postpartum.
4 Model includes outcomes at 1 and 6 months postpartum.

Appendix D. Additional Information and Data on Distributed Lag Models (DLMs)

The length of exposure/lag was in part determined by data availability; because $PM_{2.5}$ exposure assessments started 60 days before the estimated date of conception, there were a limited number days prior to the second and third trimester visits. We decided on 300 days for ease of presentation and availability of $PM_{2.5}$ data.

We tested a range of possible lag functions, including quadratic functions and natural splines with 2-5 degrees of freedom (df) and used the Akaike Information Criterion (AIC) to select the optimal one. We found that natural splines performed better; in most cases, 3 df models performed best, but in some lag length * bone combinations, 4 df was slightly better. In all cases, the resulting models look similar between three and four df so for consistency, all models shown here were modeled with 3 df.

In the first set of models, we considered all visits simultaneously in a linear mixed model. We then stratified the analyses by visit to examine the consistency of associations (including the temporal association patterns) across all visits.

DLMs were modeled using the dlnm package (v2.3.9, see https://cran.r-project.org/web/packages/dlnm/index.html).

As a sensitivity analysis, we also conducted a "naïve" analysis of daily $PM_{2.5}$ levels and bone strength measurement to visualize the temporality of the association (**Supplemental Figure 3**). In brief, we fitted linear mixed models (random intercepts, fixed effects) with all four outcome times and daily exposures (centered to the day of the visit, e.g. -1 as the day before outcome measurement), similar to the mixed models presented in the main analyses and Appendix B. We then took the coefficients from the $PM_{2.5}$ term from the models and plotted those against the lagged day with a LOESS smoothing function. We see that the results from this crude analysis and our presented DLMs are somewhat similar, providing support that our analyses adequately capture the temporal association patterns.

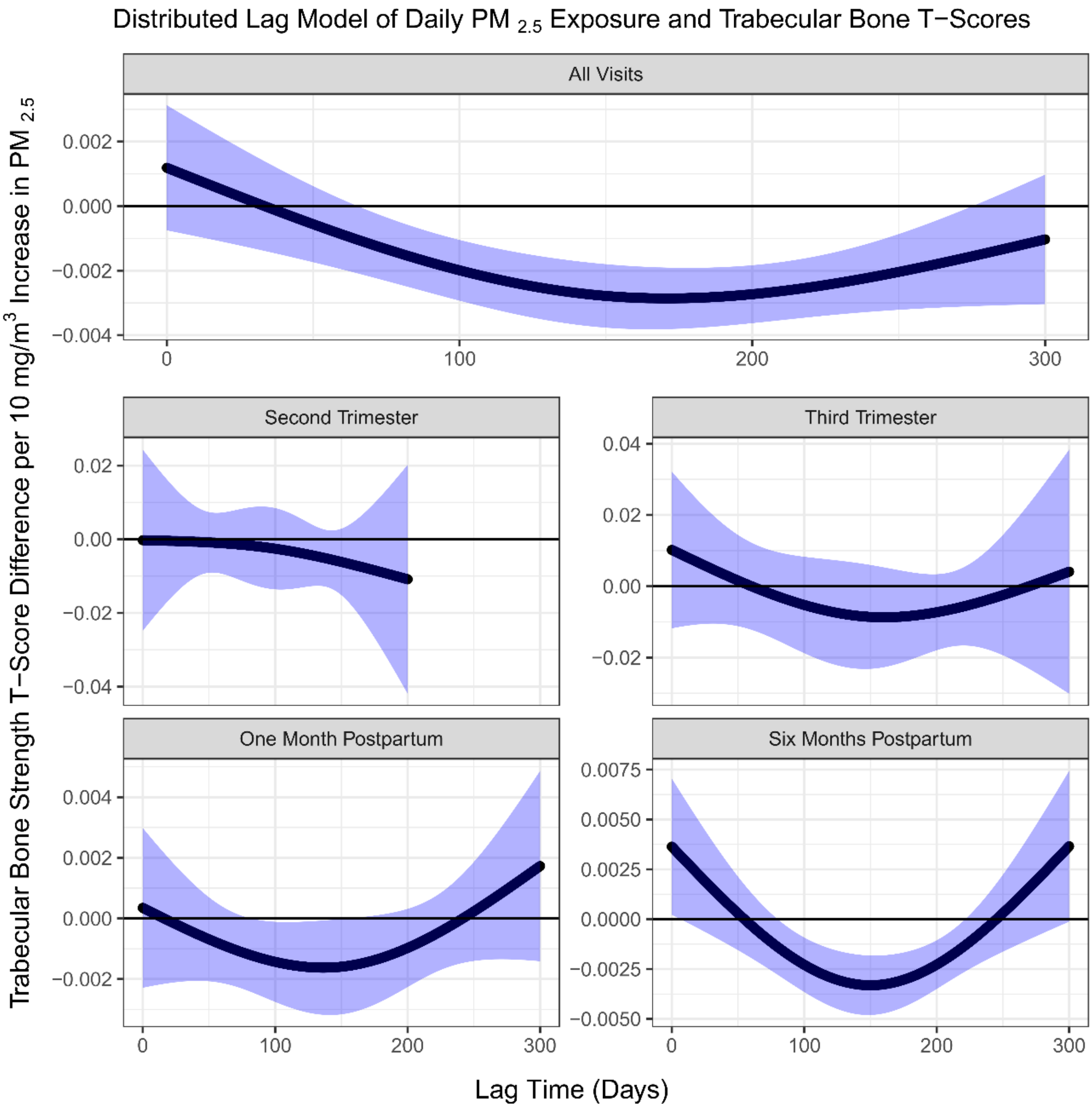

Supplemental Figure 1. Distributed lag model of trabecular bone ultrasound speed of sound t-score differences per unit increase in daily $PM_{2.5}$ exposure (µg/m^3). The results are shown for all time points and then stratified by visit (second trimester, third trimester, one month postpartum, and six months postpartum). Lag day 0 represents the day of bone ultrasound.

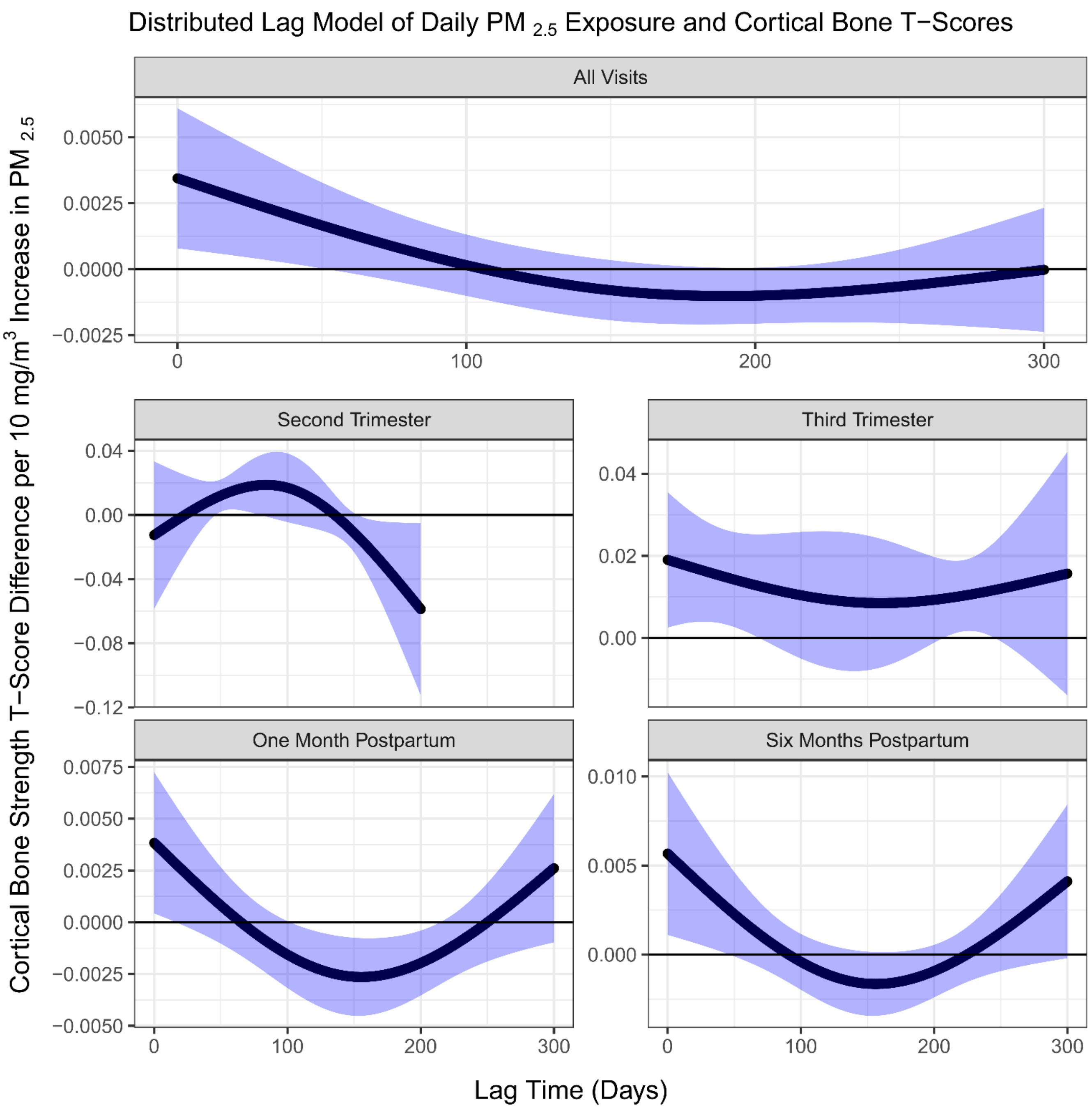

Supplemental Figure 2. Distributed lag model of cortical bone ultrasound speed of sound t-score changes per unit increase in daily $PM_{2.5}$ exposure (μg/m^3). The results are shown for all time points and then stratified by visit (second trimester, third trimester, one month postpartum, and six months postpartum). Lag day 0 represents the day of bone ultrasound.

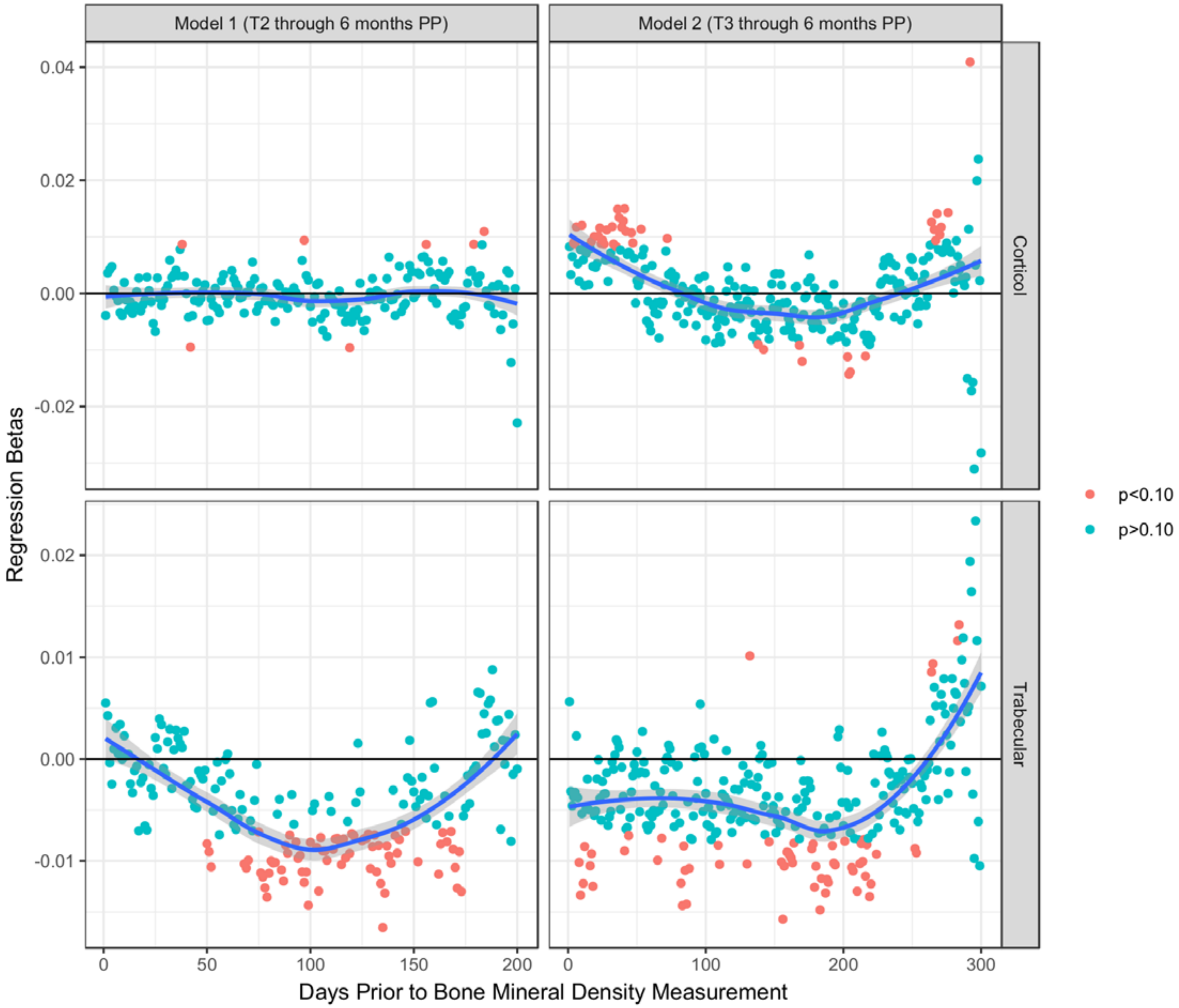

Supplemental Figure 3. Regression betas from linear models of daily $PM_{2.5}$ exposure ($\mu g/m^3$) and maternal bone strength models. Each dot represents a regression beta coefficient from a linear mixed model specifying daily exposure and repeated bone ultrasound t-score. Day 0 represents the day of bone ultrasound while days 1 represents the first day before the ultrasound. The smoothed line was estimated using a LOESS smoothing function.
